# Comparison of Cellulose Dissolution Behavior by Alkaline and Sulfuric Acid Solvents and Their Films’ Physical Properties

**DOI:** 10.3390/ma14185273

**Published:** 2021-09-13

**Authors:** Jungsoo Han, Yungbum Seo

**Affiliations:** 1 Institue of Agricultural Science, Chungnam National University, Yousung-Gu, Gung-Dong, Seoul 305-764, Korea; han1606@naver.com; 2 Department of Bio-Based Materials, Chungnam National University, Yousung-Gu, Gung-Dong, Seoul 305-764, Korea

**Keywords:** cellulose film, cellulose solvent, alkali/urea, sulfuric acid, degree of polymerization, cellulose dissolution

## Abstract

Three alkaline mixtures (NaOH/thiourea, NaOH/urea/thiourea, NaOH/urea/ZnO) and sulfuric acid were used at low temperatures as cellulose solvents, and their cellulose solubility and films’ physical properties for bleached chemical wood pulps and cotton linter were compared. Their degree of polymerization (DP) was controlled to 600–800 before dissolution. Among the alkaline solvents, NaOH/urea/ZnO gave the film the highest tensile strength and stretch. When compared to sulfuric acid, NaOH/urea/ZnO gave lower strength properties but higher crystallinity indices in the films. While alkaline solvents could not dissolve the high DP cellulose (DP ~ 2000), sulfuric acid could dissolve the high DP cellulose at below zero Celsius temperature, and the strength properties of the films were not much different from that of the low DP one. It appeared that the low-temperature sulfuric acid treatment did away with the cellulose’s DP controlling stage; it decreased cellulose DP very quickly for the high-DP cellulose at the initial stage, and as soon as the cellulose DP reached a DP low enough for dissolution, it began to dissolve the cellulose to result in stable cellulose solution.

## 1. Introduction

Bio-polymers from natural resources have gained much attention as an alternative to petroleum-based polymers due to their equivalent or better functionality, renewability, and biodegradability. Cellulose is one of the most abundant glucose-based bio-polymers available on the planet, representing about 1.5 × 10^12^ tons of the total annual biomass production [1].

It has excellent physical properties such as high elastic modulus and low coefficient of thermal expansion, as well as biodegradability and renewability [2].

Besides many practical utilization methods such as making pulp and paper, one way of using cellulose is by its dissolved form. Dissolved cellulose can be used to make a variety of products from regenerated textile fibers, packaging films, and cellulose derivatives, which are commercially produced for manufacturing advanced materials, such as separation membrane, hydrogel for bio-medical engineering, electronic devices, and various regenerated nano-fibers depending on their shapes and regeneration methods [3,4,5].

Cellulose, however, is insoluble in water and in most organic solvents due to its crystalline structure, where a high intra- and intermolecular hydrogen bond exists [6]. Recently, it has been reported that the amphiphilic nature of cellulose also contributed to its insolubility [7]. Thus, developing a powerful solvent for preparing homogeneous cellulose solutions is necessary but complicated.

Many researchers have developed powerful cellulose solvents and processes, such as the cupro process using Cu(NH_3_)_4_(OH)_2_, viscose process using NaOH, and CS_2_, DMAc/LiCl (dimethylacetamide/lithiumchloride), ionic liquid, and N-methylmorpholine-N-oxide (NMMO) [8,9,10]. Until recently, over 70% of dissolving pulp worldwide has been used in the production of viscose staples using the viscose process alone [11]. However, due to the high cost of CS_2_ and its toxicity and flammability, the viscose industry needs to employ more and more effective systems for recovering CS_2_ and H_2_S (a byproduct from the process) [9]. Regenerated cellulose fibers obtained from the NMMO system named Lyocell, with TENCEL^®^ as the brand name, are now widely used throughout the world. They have many advantages for commercialization over the viscose process, such that the recovery system of NMMO can be designed to recover >99% of the solvent and the biodegradable nature of the accidentally leaked NMMO. Cai and Zhang developed an alkali/urea aqueous solution to dissolve cellulose rapidly in 2 min at low temperature, which has a much simpler dissolution process than the other solvents [12]. The components of the solvent, such as NaOH and urea, are non-toxic and low-cost. However, cellulose pulp with a molecular weight lower than 1.14 × 10^5^ g/mol was dissolved in a 7 wt% NaOH/12 wt% urea aqueous solution.

Since then, researchers have developed NaOH/thiourea [13] and NaOH/urea/zincate aqueous solutions (NaOH/urea/ZnO) by adding zinc oxide to the existing NaOH/urea aqueous solution [14]. Jin et al. developed NaOH/urea/thiourea aqueous solution [15]. All three above alkali solvents showed higher cellulose solubility than alkali/urea solvents, but they still had the limitation of dissolving high molecular weight cellulose. Therefore, the pretreatment, such as acid hydrolysis and hot-water treatment for lowering cellulose molecular weight, as well as removing hemicellulose, was effectively applied to dissolve cellulose [11,16]. Huang et al. reported using a spruce bleached kraft pulp (BKP) with a high molecular weight (4.10 × 10^5^ g/mol), and 87.3% of α-cellulose content could be dissolved in 64 wt% sulfuric acid at low temperature; the cellulose solution could form transparent cellulose films with good mechanical properties at 2 wt% of low concentration [17]. The fact that NaOH, urea, thiourea, ZnO, and sulfuric acid are less expensive and their handling methods in real practice are well known even though their industrial process has some challenges such as equipment corrosion and a poor recovery system.

In this study, four different alkaline cellulose solvents (NaOH/urea, NaOH/thiourea, NaOH/urea/ZnO, and NaOH/urea/thiourea) and sulfuric acid were used to compare their dissolving effectiveness and the physical quality of their regenerated cellulose films. Besides, three different cellulose resources, such as cotton linter, softwood BKP, and hardwood BKP, were dissolved in an alkaline and acid solvent system to establish whether the molecular weight and the origin of the cellulose affects solvent solubility and physical properties of cellulose films.

## 2. Materials and Methods

### 2.1. Materials

The characteristics of the cellulose materials used for dissolution are summarized in Table 1. Softwood and hardwood BKP and cotton linter were used as cellulose materials with a high degree of polymerization (DP). To decrease the DP of cellulose, cotton linter and softwood BKP were irradiated with electron beam (Co-E and Sw-E in Table 1) and softwood and hardwood BKP were pretreated with dilute sulfuric acid (Sw-A and Hw-A). The electron beam irradiation energy was adjusted so that the average dosage of the beam to the samples became 20 kGy by the electron accelerator with 1.0 MeV irradiation energy (ELV-4 type, EB Tech Co., Daejeon, Korea). For dilute acid pretreatment, 1 g of softwood and hardwood BKP was treated with 20 mL 0.5 M H_2_SO_4_ at 70 °C for 90 and 180 min, respectively. DP of each cellulose sample was calculated from cellulose solution viscosity, which was measured by TAPPI standard capillary viscometer method using a 0.5 M cupriethylenediamine (CED) as a cellulose solvent (TAPPI Standard T230-om 99). The viscosities of 0.5 *w*/*v*% cellulose solutions using 0.5 M CED as a solvent determined as centipoises (cPs) were converted to DP by Equation (1) [18]. Alpha-cellulose content of each sample was measured following the TAPPI standard method T203-cm 99 (TAPPI Standard, 1999).

(1) $$DP_{W}=118.019\times \ln V^{2}+598.404\times \ln V-449.61$$

where *DP_W_* is the weight average degree of polymerization of cellulose and *V* is the CED viscosity (cPs) of 0.5 *w*/*v*% cellulose in 0.5 M CED solutions which was determined according to TAPPI Standard T230-om 99

### 2.2. Cellulose Solvent Preparation

Alkaline and sulfuric acid solvents were prepared by following the literature [12,13,14,15,17]. The solvent component ratios, pre-cooling temperatures, and cotton linter solubility values are summarized in Table 2. The solubility of cellulose in each alkaline solvent was determined by centrifugation. A total of 3.5 g of cellulose (Co-E in Table 1) was added into 100 g of each pre-cooled alkali/additive at the preset temperature, and vigorous mechanical stirring (3000 rpm) was applied for 10 min, followed by centrifugation with 1900× *g* for 10 min (MF 600, Hanil Instrument, Gimpo, Korea). After centrifugation, the settled fraction was considered insoluble fractions and was thoroughly washed using water on glass filter (G3, ~20 μm of pore size) and at 105 °C for 24 h. The solubility of cellulose in each alkaline solvent was calculated by Equation (2).

(2) $$\mathrm{Solubility}\;\left(\%\right)=\frac{W_{0}-W_{1}}{W_{0}}\times 100$$

where *W*_0_ is the weight of original cellulose, and *W*_1_ is the dried weight of settled fraction after centrifuge.

The solubility of cotton linter (Co-E in Table 1. DP 607) in NaOH/urea/water solvents was very low, and cellulose film could not be made.

### 2.3. Dissolution and Film Casting

A total of 3.5 g of cellulose with a low DP (~DP800, pretreated cellulose samples in Table 1) was added into 100 g of each alkali/additive for cellulose dissolution and centrifuged according to the already mentioned method in Section 2.2, respectively. The centrifuge was applied to remove not only the insoluble particles but also gas in the solution to obtain a homogeneous cellulose solution. The pre-calculated amount of cellulose solution was then spread onto a glass plate to form the film of 30 g/m^2^ in dried basis weight, and the glass plate was immersed immediately into the coagulation bath containing 5 wt% H_2_SO_4_ and 5 wt% Na_2_SO_4_ at room temperature for 20 min. Finally, the resulting wet film was thoroughly washed with water, separated from the glass plate, and set between filter papers to dry in the air.

In the sulfuric acid solvent system, 1.5 g of cellulose fiber with low and high DP (all cellulose samples in Table 1) was dissolved in the 100 mL of 64 wt% sulfuric acid solvent pre-cooled to −20 °C, respectively, and vigorous mechanical stirring (3000 rpm) was applied for 5 min, followed by centrifugation for removing the gas and insoluble particles (<1% of insolubility in the all cellulose samples in the sulfuric acid case). The homogeneous cellulose solution was spread onto a cold glass plate to provide the gel sheets with 30 g/m^2^ of basis weight. They were immediately immersed into the coagulation bath of 10 wt% NaOH solution at 0–4 °C for 30 min. After coagulation, wet films were thoroughly washed, separated, and set between filter papers to dry in the air.

### 2.4. Characterization

#### 2.4.1. Mechanical Properties

The mechanical properties of cellulose film were measured using universal tensile testing machine (Micro 350, Testometric Co., Ltd., Rochdale, UK). The samples were cut in strips of 10 × 60 mm and then kept at 50% of RH and 23 °C for several days before testing with 30 mm of span, 10 mm/min of test speed. Thickness of each sample was measured by using micrometer (L&W Micrometer, Lorentzen & Wettre, Stockholm, Sweden) and, the density of each film (g/cm^3^) was calculated from its basis weight (g/m^2^) divided by its thickness (μm). Breaking length is the measure of tensile strength, but it compensates basis weight differences between samples in the tensile strength measurement (ISO 1924-1, 1992). It can be calculated from the tensile strength divided by basis weight. Stretch and Young’s modulus was also measured.

#### 2.4.2. X-ray Diffraction (XRD)

The XRD pattern of samples obtained by powder XRD using a Bruker AXS D8 ADVANCE diffractometer (Bruker, Billerica, MA, USA)with CuKα radiation source (40 kV and 40 mA), 2θ = 5° and 70°, divergence angle of 0.2°, step size of 0.02. The crystallinity (CrI) of samples was determined from each XRD intensity curve (Figure S1) by using Segal’s method [19] (see Equation (3)).

(3) $$\mathrm{CrI}\left(\%\right)=\frac{I_{t}-I_{a}}{I_{t}}\times 100$$

where *I_t_* is the maximum intensity of the (200) peak for cellulose I at around 22.7° 2θ and of the (020) peak for cellulose II at around 21.7° 2θ and *I_a_* is the amorphous intensity at 18° 2θ for cellulose *I*, 16° 2θ for cellulose II, respectively [20].

#### 2.4.3. Thermogravimetric Analysis

TGA test was carried out by using TGA instrument (Mettler Toledo) with a sample weight of 4 mg, and the sample was heated from 25 °C to 500 °C at a heating rate of 10 °C/min under a nitrogen atmosphere. Thermal degradation temperatures of each sample were determined from the TGA curves (Figure S2), respectively.

#### 2.4.4. Cellulose Film Viscosity

The cellulose solvent consisting of DMAc/LiCl was used to dissolve the films, and their intrinsic viscosities were measured and calculated [9]. For the intrinsic viscosity measurement, dry weights of 0.050 g, 0.100 g, 0.150 g, and 0.200 g for each film sample were dissolved in 9 wt% DMAc/LiCl at 50 °C for 1 day after the solvent exchange [2]. The intrinsic viscosity of each sample was obtained by extrapolating the measured viscosities by using a Cannon-Fenske capillary viscometer at 30 °C.

## 3. Results

### 3.1. Comparision of Alkaline Cellulose Solvents

The physical properties of cellulose films from alkaline solvents were compared for the low DP cotton linter (see Table 3). The crystallinity and thermal degradation temperature by TGA of the cotton linter before dissolution were 64.1% and 361.4 °C, respectively, i.e., decreased considerably for the films prepared by using alkaline cellulose solvents (see Table 3). Among others, the NaOH/urea/ZnO system yielded high mechanical properties, such as high breaking length and high stretch. Yang et al. reported that the thermal stability and the tensile strength of cellulose films prepared from the NaOH/urea/ZnO solvent were higher than those prepared from the NaOH/urea solvent without ZnO, and explained that it was due to better solubility and miscibility of the cellulose in the solvent of the NaOH/urea/ZnO aqueous solution [14]. The NaOH/urea/ZnO solvent was selected to compare the physical properties of the films prepared from alkaline cellulose solvent to those of acid solvent. The NaOH/thiourea solvent made films of high Young’s modulus and high crystallinity index but low tensile strength. It was already presented by Gindl and Keckes that a high crystallinity of the cellulose materials increased their Young’s moduli [21].

### 3.2. Comparision of Alkaline and Sulfuric Acid Cellulose Solvents

Strength properties for the films prepared from NaOH/urea/ZnO and sulfuric acid solvents are shown in Table 4. Sulfuric acid gave higher breaking length and stretch than the NaOH/urea/ZnO solvent system but a lower Young’s modulus. It was also observed that the dissolution of the raw materials (cellulose fibers before dissolution) with cellulose solvents to make films decreased cellulose crystallinity greatly (more than 20% in crystallinity index), and the films prepared from the NaOH/urea/ZnO system showed higher crystallinity than those from sulfuric acid. There was a similar case in the NaOH/thiourea system in Table 3, where higher crystallinity indices of the films produced a higher Young’s moduli in the films. It was expected that the sulfuric acid solvent may break ether linkage between glucose molecules more effectively and would result in lower cellulose DP and lower tensile strength of the films even though its dissolution occurred at a very low temperature. However, higher tensile strength was observed for the films from the sulfuric acid solvent when compared to the NaOH/urea/ZnO solvent in the present study.

### 3.3. Properties of the Films Prepared from High and Low DP Cellulose

It was expected that the films from high DP cellulose (DP~2000) might allow a higher tensile strength as long as they are dissolved in alkaline or acid solvent without difficulty. However, alkaline cellulose solvent could not dissolve high DP (=2086) softwood celluloses listed in Table 1. Only with sulfuric acid, the films were prepared from both high and low DP celluloses, and their mechanical and physical properties are compared in Table 4. From Table 4, it was found that the films from the low DP cellulose gave almost identical breaking lengths to those of the high DP one within experimental error except the cotton linter case. These results were not expected. It was expected that the high DP cellulose might give longer cellulose chains after dissolution, and longer cellulose chains may allow higher strength films. The high DP cellulose gave a lower stretch but higher average values of Young’s modulus and crystallinity index in Table 4.

### 3.4. Measurement of Viscosity of the Films

DMAc/LiCl was used for the intrinsic viscosity measurement of the films prepared from softwood because the films were not dissolved well in CED. The crystallinity and intrinsic viscosity results are shown in Table 5. There were few differences in the crystallinities of the films this time. There were clear differences in intrinsic viscosity between low and high DP softwood fibers before dissolution, but only little differences between their films made by sulfuric acid dissolution. If two films had very close intrinsic viscosities, they might consist of identical size cellulose molecules. It seemed that the sulfuric acid solvent system quickly lowered the DP for the high DP cellulose initially, and later, more dissolving reactions occurred for the cellulose with lowered DP. The film viscosity from high DP cellulose converged to that of the low DP cellulose after all. If it was the case, the sulfuric acid treatment system at low temperature may remove the DP controlling stage before the dissolving process and may allow a simple cellulose dissolving system with excellent film physical properties.

## 4. Discussion

NaOH/urea/ZnO cellulose solvent gave higher film strength properties, such as higher breaking length and stretch than the other alkaline solvents. This is thought to be due to the higher stability of NaOH/urea/ZnO solvent than the other alkaline solvents such as NaOH/urea and NaOH/thiourea system, and the highly stable cellulose solution improves the cellulose molecular structure in the film during regeneration [14,15]. The films prepared from alkaline solvents gave higher Young’s moduli and crystallinity index but lower breaking lengths (Table 3) when compared to those from the sulfuric acid solvent, even though their density values were not so different. Therefore, the differences in mechanical properties of films were more related to others, including their DP and crystallinity, and were not much sensitive to the density values. For the sulfuric acid case, it can break ether linkages of the cellulose chains and may lower the cellulose DP during the cellulose dissolution simultaneously, while the alkaline solvents do not lower the cellulose DP during the dissolution process. The decrease of DP occurred in the sulfuric acid solvent system even at a low temperature of −20 °C [17]. The lowered DP cellulose by acid solvent seemed to induce low crystallinity, and the low crystallinity caused low Young’s moduli of the films [22]. The films from the sulfuric acid solvent had higher strength and stretch than those from the alkaline solvent. There were explanations that in the different solvent systems, DP of cellulose has a weaker relationship with regenerated film strength properties because other parameters such as different gel formation speed derived from different solvents during regeneration affect it more [23]. Additionally, we suggest that the amorphous structure (or lower crystallinity) of the film from the acid solvent may have caused a greater stretch, and the film from the acid solvent continued to hold the load until its tensile failure load reached beyond the tensile failure load of the films from alkaline solvents.

Another point was that the cold-temperature sulfuric acid could not dissolve the high DP cellulose, but the only reaction in that situation was mainly to lower the cellulose DP until the DP reached the point that cold sulfuric acid could dissolve it. By examining Table 5, we could confirm the mechanism; the intrinsic viscosities of the high and low DP softwood by DMAc/LiCl were quite different before film formation, but their film DPs were very close after all.

## 5. Conclusions

Three alkaline (NaOH/thiourea, NaOH/urea/thiourea, and NaOH/urea/ZnO) and sulfuric acid cellulose solvent systems were compared with respect to their solubility and film physical properties for bleached chemical pulps from softwood, hardwood, and a cotton linter, respectively.

In three alkaline solvents, NaOH/urea/ZnO gave higher film properties, such as mechanical strength, which seems to be due to the higher stability of the solvent.

Cold sulfuric acid solvent gave cellulose films greater mechanical properties than the alkaline solvents. Furthermore, the cold sulfuric acid solvent system could use high DP cellulose without pretreatment of lowering cellulose DP. Actually, the alkaline solvent system could not dissolve the high DP cellulose and should need pretreatment. It was found that in the sulfuric acid solvent system, the DP of the high DP cellulose was degraded to low DP initially until the cold sulfuric acid could dissolve the cellulose. Therefore, the DP of the films from the high and the low DP wood cellulose was not much different, and the films gave very similar film mechanical properties.

## Figures and Tables

**Table 1:** Characteristics of cellulose resources.

Sample ID	Cellulose Source	Characteristics and Pretreatment	CED Viscosity ^1^ (cPs)	DP ^2^	α-Cellulose (%)
Sw	Softwood BKP	Mixture of hemlock, douglas fir, and cedar BKP from Canada	15.6	2086	89.7
Hw	Hardwood BKP	Mixture of aspen and poplar BKP from Canada	14.6	2003	85.8
Co	Cotton linter	China first cut, pretreated with pulping and bleaching	16.6	2164	98.0
Co-E	Cotton linter	Cotton linter, electron beam treatment (20 kGy)	4.0	607	97.2
Sw-E	Softwood BKP	Softwood BKP, electron beam treatment (20 kGy)	3.8	560	85.1
Sw-A	Softwood BKP	Softwood BKP, diluted acid treatment (1 g pulp/20 mL 0.5 M H_2_SO_4_, 70°C, 90 min)	4.8	780	82.8
Hw-A	Hardwood BKP	Hardwood BKP, diluted acid treatment (1 g pulp/20 mL 0.5 M H_2_SO_4_, 70 °C, 180 min)	4.8	778	75.3

^1^ 0.5 *w/v*% cellulose/0.5 M CED solution viscosity measured from TAPPI standard T230-om 99. ^2^ Degree of polymerization.

**Table 2:** The components of cellulose solvents.

Solvent	Component Ratio (wt%)	Pre-Cooling Temperature (°C)	Solubility (%) ^2^	Ref.
NaOH/urea/water ^1^	7:12:81	−12	61.2	[12]
NaOH/urea/ZnO/water	7:12:0.5:80.5	−12	>99	[13]
NaOH/thiourea/water	9.5:4.5:86	−6	>99	[14]
NaOH/urea/thiourea/water	8:8:6.5:77.5	−6	>99	[15]
Sulfuric acid	64	−20	>99	[17]

^1^ No more use in this study due to its low dissolving capability. ^2^ 3.5 g of cotton linter (Co-E in Table 1) was used for 100 g of each solvent.

**Table 3:** Physical properties of films prepared by alkaline cellulose solvents.

Sample	Cellulose Solvent	Density (g/cm^3^)	Breaking Length (km)	Stretch (%)	Young’s Modulus (Gpa)	CrI (%)	TGA (°C)
Cotton linter, Co-E (4 cPs)	Blank	-	-	-	-	64.1	361.4
	NaOH/urea/thiourea	1.16 ± 0.23	4.88 ± 0.44	3.37 ± 0.82	2.41 ± 0.12	51.9	322.2
	NaOH/urea/ZnO	1.25 ± 0.14	5.33 ± 0.66	8.85 ± 3.53	2.89 ± 0.24	53.8	334.5
	NaOH/Thiourea	1.18 ± 0.26	4.25 ± 0.49	4.01 ± 1.80	3.30 ± 0.21	58.2	327.5

**Table 4:** Physical properties of films prepared from low DP cellulose by using NaOH/urea/ZnO and sulfuric acid as cellulose solvents.

Sample	Initial Cellulose DP	Density (g/cm^3^)	Cellulose Solvent	Breaking Length (km)	Stretch (%) (Strain to Failure)	Young’s Modulus (Gpa)	CrI (%)
Hw-A ^1^	778		-	-	-	-	72.85 ± 1.22
Sw-A ^1^	780		-	-	-	-	76.12 ± 2.31
Co-E ^1^	607		-	-	-	-	80.10 ± 2.56
Hw-A ^2^	778	1.22 ± 0.10	NaOH/urea/ZnO	3.57 ± 0.30	4.82 ± 2.32	3.33 ± 0.44	49.15 ± 3.61
Sw-A ^2^	780	1.15 ± 0.13	NaOH/urea/ZnO	3.47 ± 0.11	4.31 ± 0.64	3.66 ± 0.34	54.06 ± 2.10
Co-E ^2^	607	1.25 ± 0.14	NaOH/urea/ZnO	5.33 ± 0.66	8.85 ± 3.53	2.89 ± 0.24	53.78 ± 1.30
Hw-A ^2^	778	1.17 ± 0.12	H_2_SO_4_	4.72 ± 0.47	9.47 ± 2.00	2.78 ± 0.34	44.03 ± 1.05
Sw-A ^2^	780	1.16 ± 0.10	H_2_SO_4_	4.46 ± 0.26	6.95 ± 1.14	2.79 ± 0.12	44.87 ± 2.01
Co-E ^2^	607	1.13 ± 0.13	H_2_SO_4_	5.73 ± 0.54	10.09 ± 2.70	2.83 ± 0.33	38.48 ± 1.33
Hw ^3^	2003	1.18 ± 0.10	H_2_SO_4_	4.35 ± 0.37	3.69 ± 0.39	2.91 ± 0.24	47.70 ± 1.05
Sw ^3^	2086	1.19 ± 0.11	H_2_SO_4_	4.75 ± 0.25	4.92 ± 1.34	2.87 ± 0.28	48.70 ± 2.01
Co ^3^	2164	1.18 ± 0.10	H_2_SO_4_	4.69 ± 0.40	5.31 ± 1.80	2.98 ± 0.17	42.80 ± 1.33

^1^ Cellulosic raw materials, reduced DP by alkali (A) and electron beam (E). ^2^ Cellulose film samples prepared from reduced DP cellulose. ^3^ Cellulose film samples prepared from cellulosic raw materials.

**Table 5:** Crystallinity index and intrinsic viscosity of the high and low DP cellulose films prepared from softwood BKP fibers by using sulfuric acid.

	Initial Cellulose DP	Intrinsic Viscosity by DMAc/LiCl (mL/g)	CrI (%)
High DP softwood (Sw)	2086	757.17	79.90
Low DP softwood (Sw-E)	780	463.72	76.12
Film from high DP softwood (Sw film-H_2_SO_4_)	-	425.15	48.19
Film from low DP softwood(Sw-E film H_2_SO_4_)	-	397.86	44.38

## Data Availability

Data sharing is not applicable for this article.

## Supplementary Materials

The following are available online at https://www.mdpi.com/article/10.3390/ma14185273/s1, Figure S1: XRD curves of cellulose resources and their films prepared by alkaline and sulfuric acid solvents; Figure S2: TGA curves of electron beam (20 kGy) irradiated cotton linter (Co-E) and its cellulose films prepared by alkaline solvents, NaOH/urea/ZnO (Co-E ZnO), NaOH/thiourea (Co-E Thiourea), NaOH/urea/thiourea (Co-E NTU).
