# Supplementary material for: Comparison of Cellulose Dissolution Behavior by Alkaline and Sulfuric Acid Solvents and Their Films’ Physical Properties
Source: Materials (Basel). 2021 Sep 13;14(18):5273. doi: 10.3390/ma14185273 (PMC8465038; doi:10.3390/ma14185273)
Supplement: Supplementary file 1 [file materials-14-05273-s001.zip › materials-1343263-supplementary.pdf]

materials MDPI

Supplementary Materials

Comparison of Cellulose Dissolution Behavior by Alkaline and Sulfuric Acid Solvents and Their Films' Physical Properties

Jungsoo Han [a] and Yungbum Seo [b*]

[a] Institue of Agricultural Science, Chungnam National University, Yousung-Gu, Gung-Dong 305-764, Korea.

[b] Department of Bio-Based Materials, Chungnam National University, Yousung-Gu, Gung-Dong 305-764, Korea.

* Correspondence: ybseo@cnu.ac.kr

The supporting information documents consists 2 pages, 2 figures including XRD curves and TGA curves of samples used in this study.

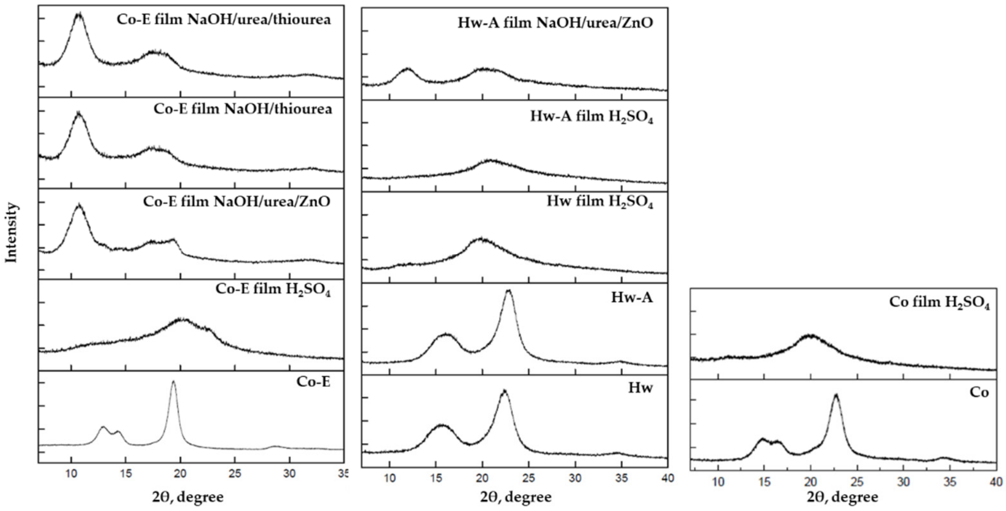

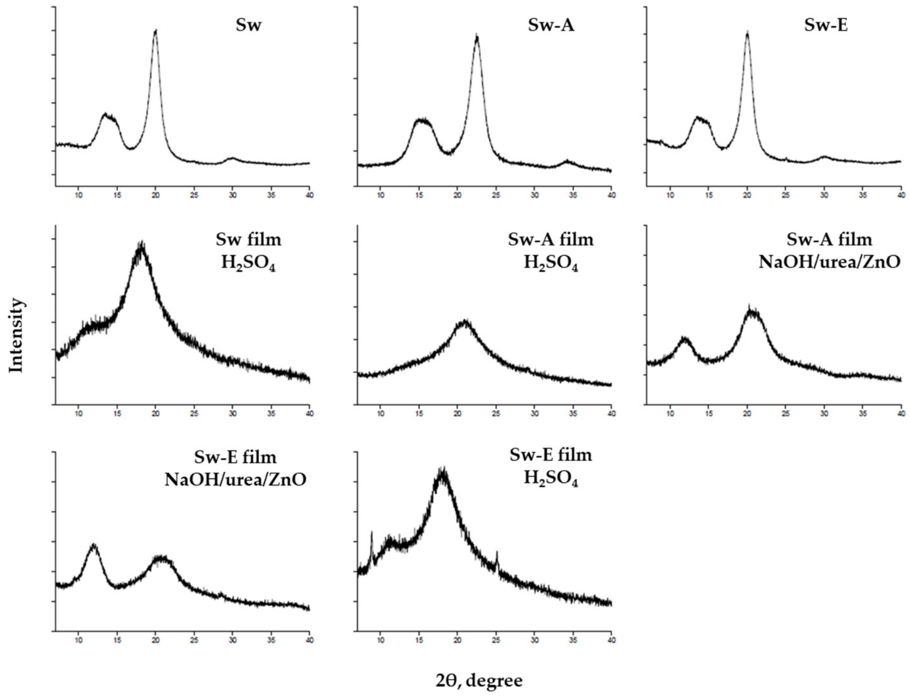

Figure S1. XRD curves of cellulose resources and their films prepared by alkaline and sulfuric acid solvents.

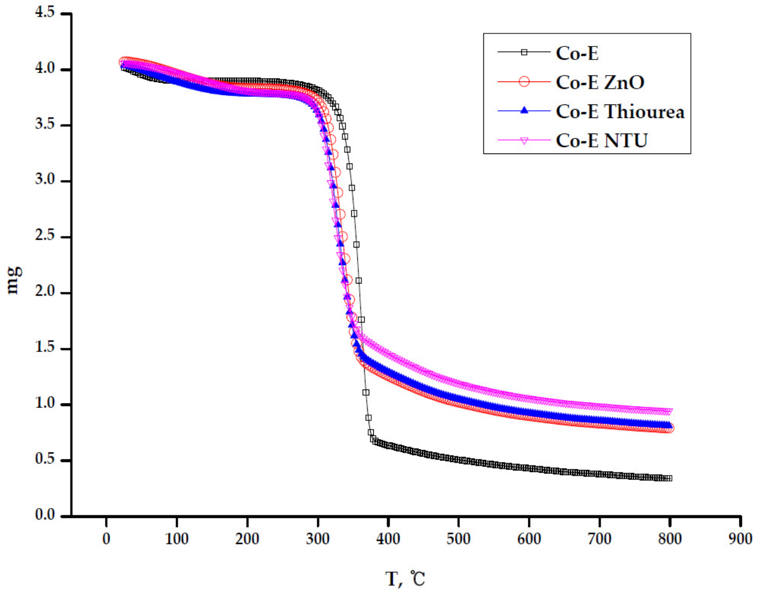

Figure S2. TGA curves of electron beam (20kGy) irradiated cotton linter (Co-E) and its cellulose films prepared by alkaline solvents, NaOH/urea/ZnO (Co-E ZnO), NaOH/thiourea (Co-E Thiourea), NaOH/urea/thiourea (Co-E NTU).
